# Supplementary material for: From sequence to enzyme mechanism using multi-label machine learning
Source: BMC Bioinformatics. 2014 May 19;15:150. doi: 10.1186/1471-2105-15-150 (PMC4229970; doi:10.1186/1471-2105-15-150)
Supplement: Additional file 2 — Java code of ml2db. Additional file ml2db_code.tar.gz contains the Java source code to run the multi-label machine learning experiments and save the results to database. The code’s Javadoc is included. [file 1471-2105-15-150-S2.zip › additional file 2/ml2db/ml2db/doc/mulan/classifier/neural/BPMLLAlgorithm.html]

BPMLLAlgorithm


JavaScript is disabled on your browser.


- Overview
- Package
- Class
- Use
- Tree
- Deprecated
- Index
- Help

- Prev Class
- Next Class

- Frames
- No Frames

- All Classes

- Summary:
- Nested |
- Field |
- Constr |
- Method

- Detail:
- Field |
- Constr |
- Method


mulan.classifier.neural

## Class BPMLLAlgorithm

- java.lang.Object
- - mulan.classifier.neural.BPMLLAlgorithm

- ---

    

  ```
  public class BPMLLAlgorithm
  extends java.lang.Object
  ```

  The implementation of Back-Propagation Multi-Label Learning (BPMLL) algorithm for neural networks.
  The algorithm uses weights decay regularization to avoid overfitting.

  Version:
  :   2012.02.27

  Author:
  :   Jozef Vilcek

  See Also:
  :   `NeuralNet`

- - ### Constructor Summary

    Constructors

    Constructor and Description

    `BPMLLAlgorithm(NeuralNet neuralNet,
    double weightsDecayCost)`

    Creates a `BPMLLAlgorithm` instance.
  - ### Method Summary

    Methods

    | Modifier and Type | Method and Description |
    | --- | --- |
    | `NeuralNet` | `getNetwork()` Returns the neural network which is learned/updated by the algorithm. |
    | `double` | `getNetworkError(double[] inputPattern, double[] expectedLabels)` Returns the error of the neural network for given input. |
    | `double` | `getWeightsDecayCost()` Returns the value of weights decay cost term used for regularization. |
    | `double` | `learn(double[] inputPattern, double[] expectedLabels, double learningRate)` Performs one learning step with given input pattern and expected output values. |

    - ### Methods inherited from class java.lang.Object

      `equals, getClass, hashCode, notify, notifyAll, toString, wait, wait, wait`

- - ### Constructor Detail


    - #### BPMLLAlgorithm

      ```
      public BPMLLAlgorithm(NeuralNet neuralNet,
                    double weightsDecayCost)
      ```

      Creates a `BPMLLAlgorithm` instance.

      Parameters:
      :   `neuralNet` - the neural network model to learn
      :   `weightsDecayCost` - the weights decay cost term used for regularization.
          The value must be greater than 0 and no more than 1.
  - ### Method Detail


    - #### getNetwork

      ```
      public NeuralNet getNetwork()
      ```

      Returns the neural network which is learned/updated by the algorithm.

      Returns:
      :   the neural network


    - #### getWeightsDecayCost

      ```
      public double getWeightsDecayCost()
      ```

      Returns the value of weights decay cost term used for regularization.

      Returns:
      :   the weights decay cost term


    - #### learn

      ```
      public double learn(double[] inputPattern,
                 double[] expectedLabels,
                 double learningRate)
      ```

      Performs one learning step with given input pattern and expected output values.
      The function outputs the error for passed input pattern.  
      The input is ignored by the algorithm (can not process) if the input example has
      assigned either all or non of the labels.
      In this case, the function returns `Double.NaN`.

      Parameters:
      :   `inputPattern` - the input pattern for the network
      :   `expectedLabels` - the ideal, expected values the network should output as a
          response for the given input. If the i-th label class belongs to the input pattern
          instance, then i-th value is +1, otherwise the value is -1.
      :   `learningRate` - the learning rate used to update the neural network weights

      Returns:
      :   the error of the network response for the passed input
          or `Double.NaN` if the passed input can not be processed.


    - #### getNetworkError

      ```
      public double getNetworkError(double[] inputPattern,
                           double[] expectedLabels)
      ```

      Returns the error of the neural network for given input. This is value of error function
      computed from network output value for given input and expected,
      ideal output for given input.  
      The input is ignored by the algorithm (can not process), if the input example has
      assigned either all or non of labels.
      In this case, the function returns `Double.NaN`.

      Parameters:
      :   `inputPattern` - the input pattern to be processed
      :   `expectedLabels` - the ideal, expected values the network should output as a
          response for the given input. If the ith label class belongs to the input pattern
          instance, then ith value is +1, otherwise the value is -1.

      Returns:
      :   the error of the network response for the passed input
          or `Double.NaN` if the passed input can not be processed


- Overview
- Package
- Class
- Use
- Tree
- Deprecated
- Index
- Help

- Prev Class
- Next Class

- Frames
- No Frames

- All Classes

- Summary:
- Nested |
- Field |
- Constr |
- Method

- Detail:
- Field |
- Constr |
- Method
